# TNF Family Cytokines Induce Distinct Cell Death Modalities in the A549 Human Lung Epithelial Cell Line when Administered in Combination with Ricin Toxin

**DOI:** 10.3390/toxins11080450

**Published:** 2019-08-01

**Authors:** Alexa L. Hodges, Cody G. Kempen, William D. McCaig, Cory A. Parker, Nicholas J. Mantis, Timothy J. LaRocca

**Affiliations:** 1 Department of Basic and Clinical Sciences, Albany College of Pharmacy and Health Sciences, Albany, NY 12208, USA; 2 Division of Infectious Disease, Wadsworth Center, New York State Department of Health, Albany, NY 12208, USA

**Keywords:** ricin, toxins, cytokines, toxin-mediated diseases, apoptosis, cathepsin, tumor necrosis factor, fas, caspases

## Abstract

Ricin is a member of the ribosome-inactivating protein (RIP) family of toxins and is classified as a biothreat agent by the Centers for Disease Control and Prevention (CDC). Inhalation, the most potent route of toxicity, triggers an acute respiratory distress-like syndrome that coincides with near complete destruction of the lung epithelium. We previously demonstrated that the TNF-related apoptosis-inducing ligand (TRAIL; CD253) sensitizes human lung epithelial cells to ricin-induced death. Here, we report that ricin/TRAIL-mediated cell death occurs via apoptosis and involves caspases -3, -7, -8, and -9, but not caspase-6. In addition, we show that two other TNF family members, TNF-α and Fas ligand (FasL), also sensitize human lung epithelial cells to ricin-induced death. While ricin/TNF-α- and ricin/FasL-mediated killing of A549 cells was inhibited by the pan-caspase inhibitor, zVAD-fmk, evidence suggests that these pathways were not caspase-dependent apoptosis. We also ruled out necroptosis and pyroptosis. Rather, the combination of ricin plus TNF-α or FasL induced cathepsin-dependent cell death, as evidenced by the use of several pharmacologic inhibitors. We postulate that the effects of zVAD-fmk were due to the molecule’s known off-target effects on cathepsin activity. This work demonstrates that ricin-induced lung epithelial cell killing occurs by distinct cell death pathways dependent on the presence of different sensitizing cytokines, TRAIL, TNF-α, or FasL.

## 1. Introduction

Ricin is a 60–65 kDa glycoprotein toxin derived from the castor bean plant, *Ricinus communis* [1,2,3]. The toxin, which presumably functions in plant defense, comprises 1–5% of the total dry weight of the bean. The cytotoxicity of ricin is based on its ability to inhibit protein synthesis in all mammalian cell types, including macrophages and epithelial cells [1,2,3]. Due to its potential to be aerosolized and deployed as a biological weapon, ricin is classified by the Centers for Disease Control and Prevention (CDC) as a select agent [1,2,3,4,5].

Technically, ricin is a member of the type II family of ribosome-inactivating proteins (RIPs), consisting of a catalytic A subunit (RTA) attached via disulfide bond to a cell-binding B subunit (RTB) [6,7]. RTB is a lectin that binds β-1,4 galactose (Gal) and N-acetylgalactosamine (GalNAc) moieties on glycolipids and glycoproteins on the surface of target cells [8]. Following binding, ricin is internalized via clathrin-dependent endocytosis and then undergoes retrograde transport to the trans Golgi network (TGN) and endoplasmic reticulum (ER) [9]. Recently, it has been shown that fucosylation and the absence of sialylation are vital for the trafficking of ricin to these compartments [9]. Once the toxin reaches the ER, the disulfide link between the A and B subunits is reduced and RTA alone is translocated into the cell cytoplasm [9,10]. RTA inhibits protein synthesis by virtue of its ability to cleave a specific glycosidic bond in the so-called sarcin-ricin loop (SRL) of rRNA in the 60s ribosomal subunit [10,11,12]. The SRL is critical for the binding of elongation factor 2 to the ribosome, which is necessary for polypeptide synthesis [13,14]. Therefore, depurination of the SRL leads to the cessation of protein synthesis [11,12]. This activity is so potent that it has been noted that a single RTA can inhibit the function of 1500 ribosomes per minute [1]. Ricin is extremely toxic following inhalation [15,16]. Wide-scale damage caused by inhaled ricin leads to acute respiratory distress syndrome (ARDS) which is characterized by a potent proinflammatory response [16,17,18,19].

Previously, we reported that the cytokine TNF-α related apoptosis-inducing ligand (TRAIL) modulates the toxicity of ricin as well as the host inflammatory response to this toxin [20]. In particular, we demonstrated that addition of TRAIL enhanced the death of Calu-3 human lung epithelial cells in a caspase-dependent manner and evoked an inflammatory response dominated by IL-6 [20]. Considering that TRAIL is one of a number of potent cell death ligands that accumulate during proinflammatory responses [21,22,23], we wanted to evaluate the cell death modulatory activities of other cytokines in the context of ricin toxicity. These cytokines include TNF-α and Fas ligand (FasL), both of which, along with TRAIL, are capable of inducing several different programmed cell death pathways [21,22,23]. In addition, proinflammatory and death-inducing cytokines such as these are abundant components in the bronchoalveolar lavage fluid of animals following ricin inhalation [24,25,26,27,28,29]. We hypothesize that lung epithelial cells compromised by ricin will be primed to undergo high levels of cell death following contact with death-inducing cytokines. We believe that this heightened cell death response to ricin will be controlled by known programmed cell death pathways. In the current study, we use biochemical approaches to provide a detailed characterization of A549 human lung epithelial cell death responses to ricin administered in combination with TRAIL, TNF-α, or FasL. Defining these cell death responses and identifying multiple steps at which they can be inhibited may lead to new therapeutic approaches to ricin toxicity targeted against specific programmed cell death pathways.

## 2. Results

### 2.1. Ricin-Induced Death Is Primed by TRAIL, TNF-α, and FasL

**Figure 1:**
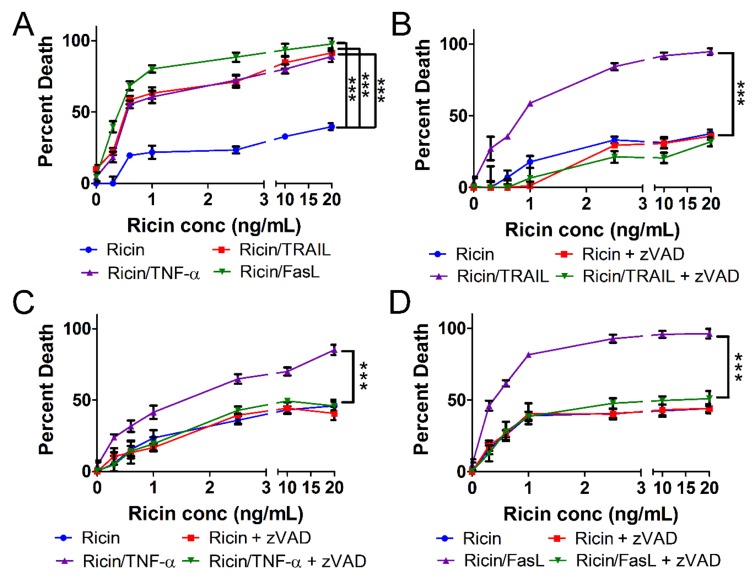
Extrinsic cytokines enhance ricin-induced death of A549 cells in a zVAD inhibitable manner. A549 lung epithelial cells were treated with ricin alone or in combination with 100 ng/mL TRAIL, TNF-α, or FasL for 24h at 37 °C followed by measurement of cell death via WST-1 assay. (**A**) All 3 cytokines/ligands resulted in a significant increase in cell death relative to treatment with ricin alone. A549 cell death induced by ricin combined with (**B**) TRAIL, (**C**) TNF-α, or (**D**) FasL is prevented by the pan-caspase inhibitor, zVAD-fmk (50 μM). Results are the average of 3 independent experiments. Error bars = standard deviation. Two-way analysis of variance (ANOVA), *** *p* < 0.001.

To test the hypothesis that extrinsic cytokines cause an increase in ricin-induced cell death, A549 cells were treated with increasing doses of ricin in the absence or presence of 100 ng/mL TRAIL, TNF-α, or FasL for 24 h at 37 °C. Over a range of toxin concentrations, addition of each of the cytokines resulted in a significant increase in ricin-induced cell death (Figure 1A). Our previous work indicated that ricin/TRAIL induces apoptosis of Calu-3 human lung epithelial cells [20]. Indeed, when we used the pan-caspase inhibitor zVAD-fmk, A549 cell death induced by ricin combined with TRAIL, TNF-α, or FasL was prevented (Figure 1B−D). Collectively, the results of Figure 1 indicate that TRAIL, TNF-α, and FasL enhance ricin-induced cell death in a manner that is likely caspase-dependent apoptosis.

### 2.2. Cell Death Induced by Ricin/TRAIL Is Associated with Caspase Activation while Death by Ricin/TNF-α and Ricin/FasL Is Not

**Figure 2:**
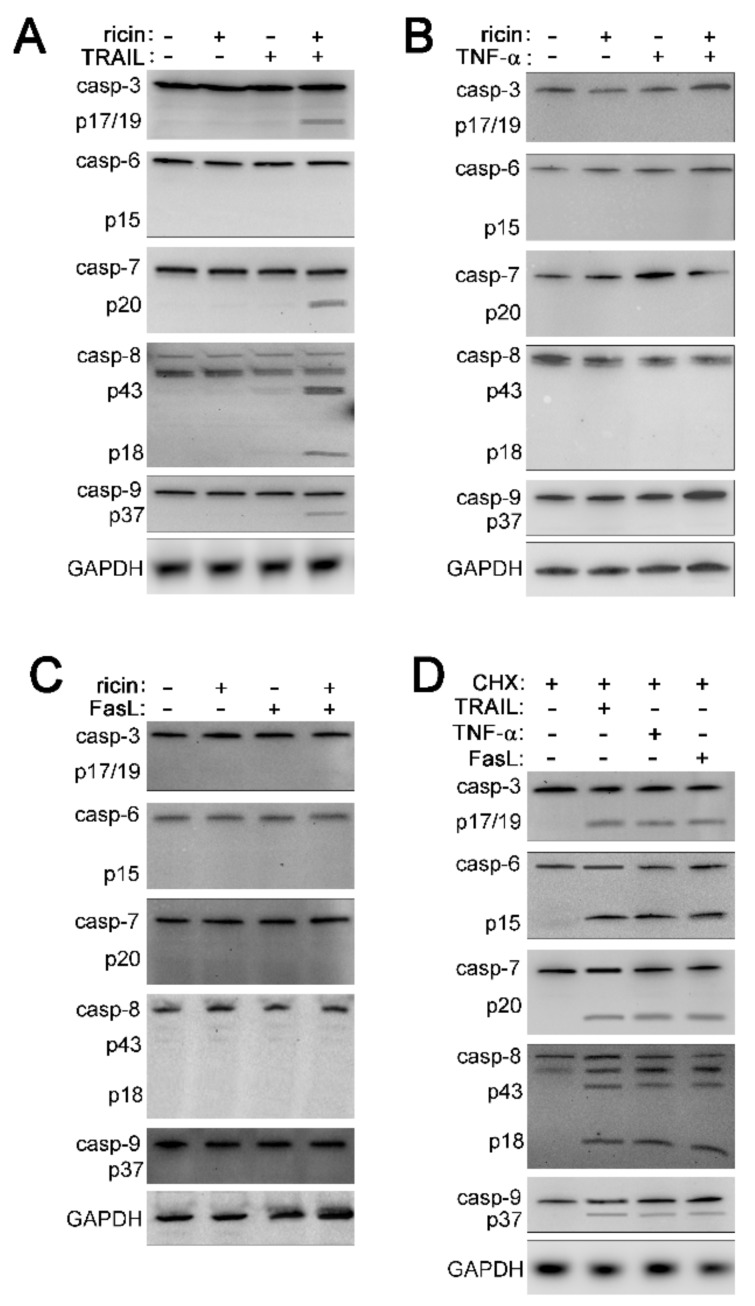
The combination of ricin and TRAIL induces caspase activation, while addition of TNF-α or FasL with ricin does not. A549 lung epithelial cells were treated with 1 ng/mL ricin alone or in combination with 100 ng/mL TRAIL, TNF-α, or FasL for 4 h at 37 °C followed by cell lysis and western blot. (**A**) The combination of ricin and TRAIL results in cleavage/activation of caspases-3, -7, -8, and -9. Caspase-6 is not cleaved/activated in response to ricin/TRAIL (**B**,**C**) The combination of ricin/TNF-α or ricin/FasL does not result in the cleavage/activation of caspases. (**D**) A549 cells were treated with 250 ng/mL cycloheximide (CHX) combined with TNF-α, FasL, or TRAIL as a positive control for TRAIL-, TNF-, and FasL-induced apoptosis. As expected, when cycloheximide is combined with TNF-α, FasL, or TRAIL it results in the cleavage/activation of caspases-3, -6, -7, -8, and -9. Shown are representative blots from 3 independent experiments.

To get a clear view of the involvement of caspases, the effectors of apoptosis [21], in cell death by ricin combined with TRAIL, TNF-α, or FasL, A549 cells were treated with 1 ng/mL ricin combined with 100 ng/mL TRAIL, TNF-α, or FasL for 4 h at 37 °C followed by cell lysis and western blot. Treatment with ricin or any of the cytokines alone did not result in caspase cleavage/activation (Figure 2A–C). When combined with TRAIL, ricin induced cleavage/activation of caspases-3, -7, -8, and -9 but not caspase-6 (Figure 2A and Figure S1). However, the combination of ricin and TNF-α or ricin and FasL did not cause cleavage/activation of any caspase tested (Figure 2B,C). To determine if caspases were cleaved with slower kinetics, we measured caspase cleavage in response to ricin/TNF-α or ricin/FasL after 8 h of treatment. However, caspases were not cleaved/activated in A549 cells at this time point (Figure S2). As a positive control for TRAIL-, TNF-, and FasL-induced apoptosis, A549 cells were treated with 250 ng/mL cycloheximide (CHX) combined with 100 ng/mL TRAIL, TNF-α, or FasL [30,31,32,33,34,35,36,37]. Apoptosis induced by CHX combined with any of the cytokines resulted in cleavage/activation of caspases-3, -6, -7, -8, and -9 (Figure 2D and Figure S3). These results clearly demonstrate that caspases are activated following attack by ricin/TRAIL but are not affected by ricin/TNF-α and ricin/FasL.

### 2.3. The Combination of Ricin and TRAIL Induces Caspase-Dependent Apoptosis

**Figure 3:**
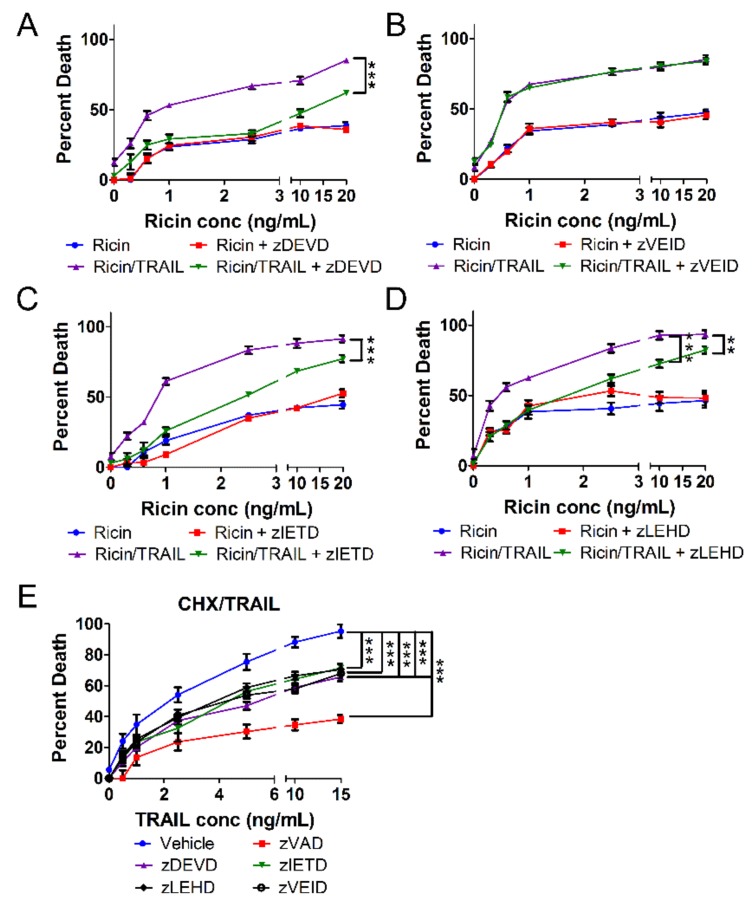
A549 cell death induced by ricin/TRAIL depends on caspases-3, -7, -8, and -9. A549 lung epithelial cells were treated with ricin alone or in combination with 100 ng/mL TRAIL in the presence or absence of caspase inhibitors for 24 h at 37 °C followed by measurement of cell death via WST-1 assay. Cell death induced by the combination of ricin and TRAIL was prevented by inhibition of (**A**) caspases-3 and -7 with zDEVD-fmk (30 μM) but not (**B**) caspase-6 with zVEID-fmk (30 μM). Moreover, cell death by ricin/TRAIL was prevented by inhibitions of (**C**) caspase-8 with zIETD-fmk (30 μM) and (**D**) caspase-9 with zLEHD-fmk (10 μM). (**E**) A549 cells were treated with the combination of 250 ng/mL cycloheximide (CHX) and TRAIL as a positive control for TRAIL-induced apoptosis. As expected, cycloheximide/TRAIL-induced apoptosis is prevented by all caspase inhibitors tested. Results are the average of 3 independent experiments. Error bars = standard deviation. Two-way ANOVA, *** *p* < 0.001, ** *p* < 0.01.

The results of Figure 2 suggest that ricin/TRAIL causes activation of caspases, and thus apoptosis, while ricin/TNF-α and ricin/FasL do not. This finding was unexpected for ricin/TNF-α and ricin/FasL. Therefore, we decided to perform a detailed characterization of A549 cell death caused by ricin combined with TRAIL, TNF-α, or FasL. A549 cells were treated with increasing doses of ricin and 100 ng/mL TRAIL for 24 h at 37 °C in the absence or presence of specific caspase inhibitors. We determined that cell death induced by ricin combined with TRAIL depends on executioner caspases-3 and -7 (Figure 3A and Figure S4) as well as initiator caspases-8 and -9 (Figure 3C,D and Figure S4) but not caspase-6 (Figure 3B). These results are in agreement with our caspase activation/cleavage results of Figure 2. As a positive control for TRAIL-induced apoptosis, A549 cells were treated with 250 ng/mL CHX combined with increasing concentrations of TRAIL [30] (Figure 3E). Cell death induced by CHX/TRAIL was prevented by inhibition of caspases-3, -7, -8, and -9 as the combination of CHX and TRAIL induces apoptosis [30,38,39]. These results indicate that ricin induces caspase-dependent apoptosis of human lung epithelial cells when combined with TRAIL similar to the combination of CHX and TRAIL.

### 2.4. Ricin Combined with TNF-α or FasL Induces Caspase-Independent Cell Death

**Figure 4:**
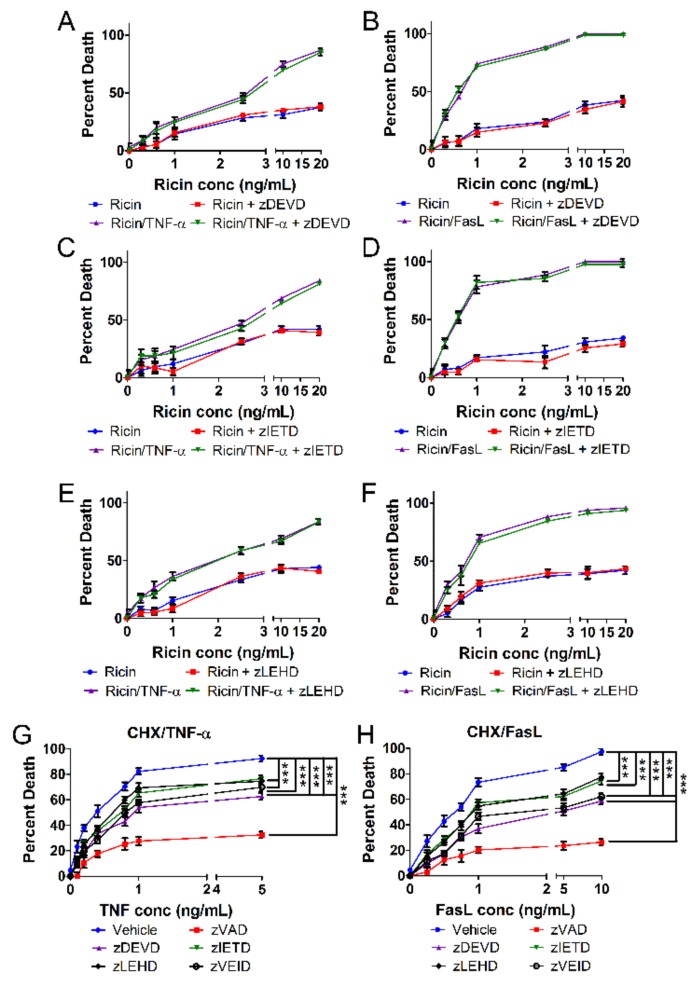
Ricin/TNF-α and ricin/FasL both induce caspase-independent death of A549 cells. A549 lung epithelial cells were treated with ricin alone or in combination with 100 ng/mL TNF-α or FasL in the presence or absence of caspase inhibitors for 24 h at 37 °C followed by measurement of cell death via WST-1 assay. Cell death induced by ricin/TNF-α and ricin/FasL was not prevented by inhibition of (**A**,**B**) caspases-3 and -7 with zDEVD-fmk (30 μM), (**C**,**D**) caspase-8 with zIETD-fmk (30 μM), or (**E**,**F**) caspase-9 with zLEHD-fmk (10 μM) (**G**,**H**) A549 cells were treated with the combination of 250 ng/mL cycloheximide (CHX) and TNF-α or cycloheximide and FasL as positive controls for TNF- and FasL-induced apoptosis. As expected, cycloheximide/TNF- and cycloheximide/FasL-induced apoptosis is prevented by all caspase inhibitors tested. Results are the average of 3 independent experiments. Error bars = standard deviation. Two-way ANOVA, *** *p* < 0.001.

We next characterized A549 cell death by ricin/TNF-α or ricin/FasL with respect to the findings of Figure 2. A549 cells were treated with increasing doses of ricin and 100 ng/mL TNF-α or FasL for 24 h at 37 °C. Cell death induced by ricin/TNF-α or ricin/FasL was not prevented by specific inhibition of caspases-3, -6, -7, -8, or -9 (Figure 4A–F and Figure S5). In contrast to the results with zVAD-fmk (Figure 1C,D), these results are in agreement with those of Figure 2. As positive controls for TNF- and FasL-induced apoptosis, A549 cells were treated with 250 ng/mL CHX combined with increasing concentrations of TNF-α [30,31,32,33,34,35,36,37] (Figure 4G) or FasL [30,34,36] (Figure 4H). TNF- and FasL-induced apoptosis were prevented by inhibition of caspases-3, -7, -8, and -9 (Figure 4G,H). These results indicate that ricin/TNF-α and ricin/FasL induce caspase-independent cell death which is distinct from the caspase-dependent apoptosis induced by CHX/TNF-α and CHX/FasL.

**Figure 5:**
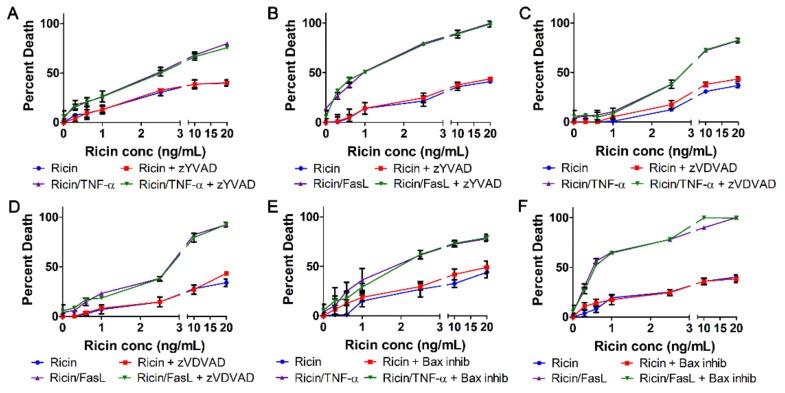
A549 cell death induced by ricin in combination with TNF-α or FasL does not depend on caspase-1, -2, or Bax. A549 lung epithelial cells were treated with ricin alone or in combination with 100 ng/mL TNF-α or FasL in the presence or absence of various pharmacologic inhibitors. Cell death induced by the combination of ricin with TNF-α or FasL was not prevented by inhibition of (**A**,**B**) caspase-1 with zYVAD-fmk (10 μM), (**C**,**D**) caspase-2 with zVDVAD-fmk (50 μM), or (**E**,**F**) Bax with a peptide-based inhibitor (Bax-inhibiting peptide v5, 100 μM). Results are the average of 3 independent experiments. Error bars = standard deviation. Two-way ANOVA, *** *p* < 0.001.

Since cell death induced by ricin/TNF-α and ricin/FasL was prevented by the pan-caspase inhibitor, zVAD-fmk but not inhibition of specific apoptotic caspases (Figure 1C,D, Figure 4 and Figure S5), we investigated the involvement of alternative caspases and apoptosis effectors in both instances of cell death. Inhibition of caspase-1, the central effector caspase of pyroptosis [40,41], did not prevent cell death by ricin/TNF-α or ricin/FasL (Figure 5A,B). Caspase-2 is thought to have initiator and effector roles in some forms of apoptosis [42] yet its inhibition did not prevent cell death by ricin combined with either cytokine (Figure 5C,D). During intrinsic apoptosis, Bax is critical for mitochondrial outer membrane pore formation and cytochrome *c* release [21]. However, when Bax was inhibited there was no effect on the cell death induced by ricin/TNF-α or ricin/FasL (Figure 5E,F) in contrast to its effect on apoptosis induced by CHX/TNF-α or CHX/FasL (Figure S6). Furthermore, cytochrome *c* was not observed in the cytoplasm of cells treated with ricin/TNF-α or ricin/FasL (Figure S7).

Necroptosis is another major pathway of cell death in addition to apoptosis which may be induced by TNF-α and FasL [23,43]. Thus, we investigated the involvement of RIP1, the initiator kinase of necroptosis [23,43], in cell death by ricin combined with TNF-α or FasL. Cell death induced by ricin combined with either cytokine was not prevented by the RIP1 inhibitor, necrostatin-1s (Figure S8). Collectively, the results of Figure 5 and Figure S7 indicate that ricin/TNF-α and ricin/FasL do not induce one of the classical non-apoptotic cell death pathways. Additionally, the pattern of Mcl1 protein loss does not differ between ricin/TRAIL vs. ricin/TNF-α or ricin/FasL in A549 cells (Figure S9). Thus, we believe the distinct cell death responses to ricin combined with different cytokines is not the result of altered kinetics of protein synthesis inhibition.

### 2.5. Cell Death Induced by Ricin/TNF-α and Ricin/FasL Depends on Cathepsins

**Figure 6:**
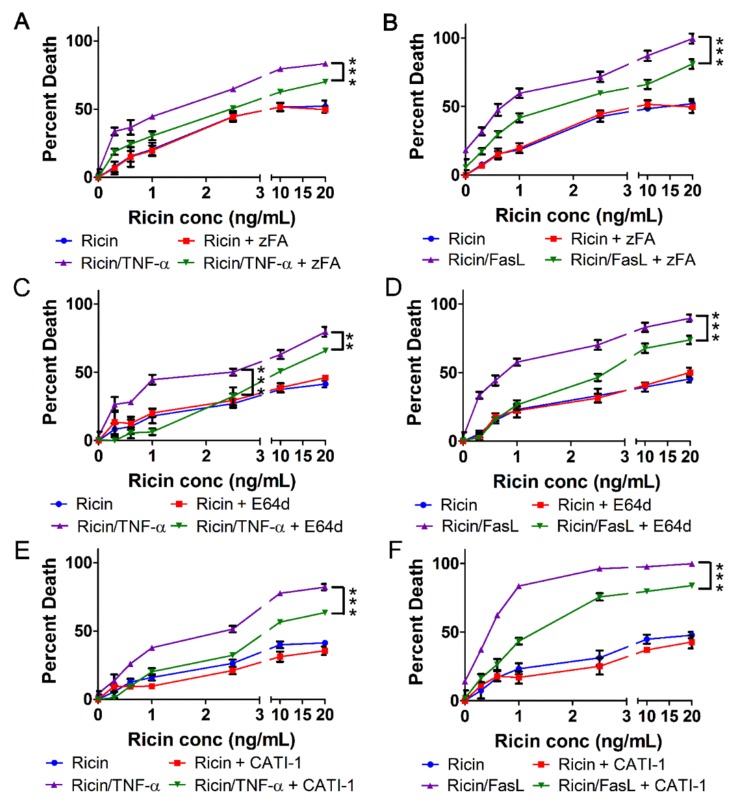
Cell death induced by ricin/TNF-α or ricin/FasL depends on cathepsins. A549 lung epithelial cells were treated with ricin alone or in combination with 100 ng/mL TNF-α or FasL for 24 h at 37 °C followed by measurement of cell death via WST-1 assay. Cell death induced by ricin/TNF-α and ricin/FasL was partially prevented by inhibition of cathepsins with (**A**,**B**) zFA-fmk (50 μM), (**C**,**D**) E64d (50 μM), or (**E**,**F**) cathepsin inhibitor 1 (CATI-1, 20 μM). Results are the average of 3 independent experiments. Error bars = standard deviation. ANOVA, *** *p* < 0.001, ** *p* < 0.01.

Treatment with zVAD-fmk (pan-caspase inhibitor) prevented cell death induced by ricin/TNF-α and ricin/FasL (Figure 1C,D) yet it does not appear as though caspases are activated during these instances of cell death (Figure 2, Figure 4, Figure S2 and Figure S5). Importantly, zVAD-fmk may have off-target effects against cathepsins in addition to its specific effects on caspases [44,45]. Therefore, we wondered if the effects of this inhibitor were due to deactivation of cathepsins. Interestingly, zFA-fmk, an inhibitor of executioner caspases-2, -3, -6, and -7 [46] as well as cathepsins B, L and S [47], caused significant inhibition of cell death induced by ricin combined with either TNF-α or FasL (Figure 6A,B). In addition, E64d, an inhibitor of calpains as well as cathepsins B, H, and L [48], blunted A549 cell death by ricin/TNF-α or ricin/FasL (Figure 6C,D). However, calpeptin, an inhibitor of calpains as well as cathepsins L and K [49], had no effect on cell death by ricin/TNF-α or ricin/FasL (Figure S10). Cathepsin inhibitor 1 (CATI-1) has activity against cathepsins L and S with cathepsin B being its primary target [50]. Inhibition with CATI-1 prevented A549 cell death by ricin combined with TNF-α or FasL (Figure 6E,F). Importantly, CATI-1 had not effect on apoptosis induced by ricin/TRAIL (Figure S11). Cathepsin-dependent cell death is often associated with a dependence on reactive oxygen species (ROS) [51]. We investigated the involvement of ROS in cell death induced by ricin/TNF-α and ricin/FasL using the antioxidant, N-acetylcysteine. Scavenging of ROS by N-acetylcysteine (NAC) resulted in a significant prevention of cell death induced by ricin/TNF-α or ricin/FasL (Figure S12). Collectively, these results indicate that when combined with TNF-α or FasL, ricin induces cell death that depends on cathepsins with a likely role for ROS.

The dominant cell line used in this study was A549 human lung epithelial cells, a cell line derived from lung carcinoma. These cells were chosen as they represent a model of human alveolar, type II pneumocytes for drug and toxin metabolism [52,53]. To determine if our results were unique to A549 cells, we conducted experiments with Calu3 human lung epithelial cells. Previously, we showed that ricin/TRAIL induces caspase-dependent apoptosis in Calu3 cells [20]. Here we show that Calu3 cells appear to be insensitive to cell death by ricin/TNF-α or ricin/FasL (Figure S13). Therefore we tested another human cell line, U937 monocytes, as alveolar macrophages are another target cell of ricin upon inhalation. Indeed, ricin/TRAIL induced caspase-dependent apoptosis in U937 cells while ricin/TNF-α and ricin/FasL induced cathepsin-dependent cell death (Figure S14). While the results obtained in U937 cells supports our findings in A549 cells, these are also a cell line derived from cancerous tissue. Future work should focus on comparing these findings to those obtained in primary lung epithelial cells and macrophages.

## 3. Discussion

### 3.1. Distinct Cell Death Modalities

Our results indicate that ricin induces distinct cell death modalities in A549 human lung epithelial cells depending upon the TNF family cytokine with which it is paired. As ricin is the common factor in each of these scenarios, it is tempting to speculate that the cytokines administered with ricin and their downstream signaling pathways are the primary factors in producing these distinct outcomes. However, when combined with CHX, another molecule that inhibits protein synthesis [54], TRAIL, TNF-α, and FasL all induced caspase-dependent apoptosis (Figure 3E and Figure 4G–H). Therefore, there is something unique about the combination of ricin with each of these cytokines which produces different cell death responses. While ricin and CHX both inhibit protein synthesis, they do so by distinct mechanisms: ricin via depurination of rRNA [10,11,13,14] and CHX via occupation of the ribosomal E site [54]. It is possible that these fine mechanistic differences account for the distinct outcomes of cell death depending upon whether TNF family cytokines are paired with ricin or CHX. In addition, there is evidence that the protein synthesis inhibition activity of ricin does not correlate with execution of cell death in other systems [55]. The fact that the cell death caused by ricin does not correlate with protein synthesis inhibition may also provide an explanation for the difference in these cell death outcomes. While TRAIL, TNF-α, and FasL have common upstream signaling events, each pathway eventually diverges [56,57]. Thus, there is precedent for induction of distinct cell death pathways in response to TRAIL, TNF-α, and FasL. The final intersection point in these signaling pathways is likely the death-inducing signaling complex (DISC) [58,59]. We speculate that following DISC formation the signaling pathway induced by ricin/TRAIL diverges from that induced by ricin/TNF-α and ricin/FasL accounting for these distinct cell death outcomes. Future work will include a detailed study on these signaling responses to address this.

Of note is the fact that ricin alone did not appear to induce caspase-dependent apoptosis in A549 cells throughout this work. This is in contrast to in vivo findings as well as results from other human cell types [60,61]. In HeLa cervical cells, MCF7 mammary cells, and U937 monocytes, ricin alone induces caspase-dependent apoptosis [60,61]. The cell death that ricin induces in human lung epithelial cell lines is less certain. The inability of ricin alone to induce apoptosis in A549 cells does not appear to be a unique feature of this cell line as we obtained similar results in Calu3 lung epithelial cells [20]. Therefore, while ricin induces apoptosis in other human cell types, lung epithelial cells may exhibit a different cell death modality in response to this toxin.

### 3.2. Cathepsin-Dependent Cell Death

We originally hypothesized that the combination of ricin with TRAIL, TNF-α, or FasL would produce caspase-dependent apoptosis. However, this was only the case when ricin was combined with TRAIL (Figure 2A and Figure 3). The fact that the pan-caspase inhibitor, zVAD-fmk, prevented cell death by ricin/TNF-α and ricin/FasL suggested that, like TRAIL, these cytokines also provoke caspase-dependent apoptosis when administered with ricin (Figure 1C,D). However, it is clear that there is no role for caspases in cell death by ricin/TNF-α or ricin/FasL (Figure 4A–F and Figure S5). Moreover, these signaling molecules remain inactivated in response to ricin/TNF-α and ricin/FasL (Figure 2B,C and Figure S2). In addition, we confirmed that alternative caspases (caspases-1 and -2) and downstream apoptosis effectors (Bax) do not play a role in cell death by ricin/TNF-α or ricin/FasL (Figure 5).

Cathepsins are proteases originally discovered to be resident to lysosomes but are present in the cytoplasm and nucleus as well [62]. These molecules may have roles in apoptosis, necroptosis, and pyroptosis [51,62,63]. The results presented in Figure 6 clearly indicate that ricin/TNF-α and ricin/FasL each induce cathepsin-dependent death of A549 human lung epithelial cells. However, we believe that this is a distinct cathepsin-driven cell death pathway and not a component of another cell death pathway (e.g., apoptosis, necroptosis, etc.). Indeed, we ruled out contributions from apoptosis (Figure 2B,C and Figure 4), necroptosis (Figure S2), and pyroptosis (Figure 5A,B) to cell death induced by ricin/TNF-α and ricin/FasL. Cathepsin-driven cell death is often accompanied by ROS activity [51]. Therefore, our findings that N-acetylcysteine (NAC) prevents cell death by ricin/TNF-α or ricin/FasL (Figure S4) are consistent with known mechanisms of cathepsin-dependent cell death, particularly since we have ruled out contributions from apoptosis, necroptosis, and pyroptosis (Figure 4, Figure 5A,B and Figure S8). It is possible that the cathepsin-dependent cell death induced by ricin/TNF-α and ricin/FasL is a form of lysosome-dependent cell death, which requires the release of cathepsins from lysosomes via permeabilization [51,63]. However, cathepsins are known to be resident to the cytoplasm as well [62], making lysosomal involvement in these pathways unclear. If lysosomal permeabilization is the basis for cathepsin involvement in these pathways, it is likely to be ROS-mediated, particularly in light of the results from Figure S12. The other major stimulus for lysosomal permeabilization is Bak/Bax [51], whose involvement we have ruled out (Figure 5E,F).

Interestingly, in Figure S11 NAC did not prevent cell death induced by ricin alone. This is in contrast to other reports that ROS are involved in cell death induced by ricin [64,65]. Data on the involvement of ROS in ricin-induced death of human lung epithelial cells are lacking, however. As noted earlier, ricin alone clearly induces caspase-dependent apoptosis in human monocytes, mammary cells, and cervical cells [60,61]. However, in this work as well as our prior work we have shown that ricin alone does not induce caspase-dependent apoptosis of human lung epithelial cells lines A549 (Figure 2, Figure 3 and Figure 4) and Calu3 [20]. We hypothesize that NAC did not affect cell death by ricin alone in A549 cells as these cells undergo a cell death response to ricin which differs from the caspase-dependent apoptosis that ricin induces in other human cell types. This also agrees with the accepted concept that ROS have a key role in apoptosis [66].

### 3.3. Therapeutic Implications

Enhancement of ricin-induced cell death by TNF family cytokines presents new potential therapeutic targets against ricin toxicity. Targeting these cell death ligands directly with neutralizing antibodies represents a viable therapeutic option as does targeting ricin with neutralizing antibodies. We explored this previously at the cellular level [20]. Our identification of activated cell death pathways in response to ricin combined with TNF family cytokines reveals further potential therapeutic targets (i.e., specific components of each cell death pathway). However, the finding that different cytokines induce distinct cell death pathways in combination with ricin suggests that a combinatorial approach may be best in the context of ricin toxicity. Rather than targeting apoptosis, which would limit ricin/TRAIL-induced cell death only, both caspase-dependent apoptosis and cathepsin-dependent cell death should be targeted. In addition, we cannot rule out contributions from other cytokines and cell death pathways to ricin-induced cell death in the in vivo setting. This could result in the targeting of multiple cell death pathways at various steps to limit ricin-induced toxicity. Another potential therapeutic implication of this work may extend to the targeting of tumors, as we have utilized cell lines derived from cancerous tissue throughout this research. Recent research on cancer therapeutics has partly focused on transfection of cytotoxic genes into tumor cells [67]. Ricin may serve as a good candidate for such targeted cancer therapy, particularly if combined with administration of TNF family cytokines.

## 4. Materials and Methods

### 4.1. Reagents and Inhibitors

Ricin toxin (*Ricinus communis* agglutinin II) was purchased from Vector Laboratories and used at the concentrations noted. Ricin was dialyzed in 1× PBS using 10K MW-cutoff Slide-A-Lyzer dialysis cassettes (Pierce, Rockford, IL, USA) prior to experimentation. Cycloheximide (Sigma, St. Louis, MO, USA) was used at a concentration of 500 ng/mL unless noted otherwise. Recombinant human TRAIL (Peprotech, Rocky Hill, NJ, USA), TNF-α (Shenandoah Biotechnology, Warwick, PA, USA), and FasL (Super Fas ligand, Enzo Life Sciences, Farmingdale, NY, USA) were used at a concentration of 100 ng/mL unless noted otherwise. All caspase inhibitors are irreversible and were purchased from ApexBio (Houston, TX, USA). Pan-caspase inhibitor zVAD-fmk, executioner caspase and cathepsin inhibitor zFA-fmk, and caspase-2 inhibitor zVDVAD were each used at a concentration of 50 μM. Caspase-3/7 inhibitor zDEVD, caspase-8 inhibitor zIETD, and caspase-6 inhibitor zVEID were each used at a concentration of 30 μM. Caspase-1 inhibitor zYVAD-fmk and caspase-9 inhibitor zLEHD-fmk were each used at a concentration of 10 μM. Necrostatin-1s (EMD Millipore, Burlington, MA, USA) was used at a concentration of 50 μM. Bax-inhibiting peptide v5 (EMD Millipore) was used at a concentration of 100 μM. E64d was used at a concentration of 10 μM. Cathepsin inhibitor 1 (CATI-1, ApexBio) was used at a concentration of 20 μM. N-acetylcysteine (Sigma) was used at a concentration of 10 mM.

### 4.2. Cell Culture

A549 lung epithelial cells (CCL-185, ATCC, Manassas, VA, USA) were cultured in Ham’s F-12K medium (Thermo Fisher) with 10% FBS as recommended by the ATCC. Cells were grown in a humidified incubator with 5% CO_2_ at 37 °C. Cells were cultured in 75-cm^2^ cell culture flasks and subcultured when they reached ~80% confluence at a 1:5 dilution. Cells were lifted using TrypLE Express (Thermo Fisher, Waltham, MA, USA) at 37 °C. Cells were maintained at a maximum of 10 passages for the experiments in this work.

### 4.3. Cell Death Assays

A549 lung epithelial cells were seeded at 1200 cells/well in 96-well plates (Celltreat, Pepperell, MA, USA) and allowed to culture for 24 h in Ham’s F-12K medium (Thermo Fisher) with 10% FBS (VWR, Radnor, PA, USA) at 37 °C and 5% CO_2_. Following this, cells were washed and the following was added to wells in Ham’s F-12K medium: (1) ricin, (2) 100 ng/mL cell death ligand/cytokine (TRAIL, TNF-α, or FasL), or (3) ricin combined with 100 ng/mL cell death ligand/cytokine. Cells in negative control wells were treated with media alone. In some experiments (Figure 1B), CHX was used in place of ricin. In experiments where inhibitors were used, they were added to cells 1 h before the addition of ricin and cell death ligands/cytokines. Appropriate vehicle controls were used for each inhibitor. After the addition of ricin and cell death ligands/cytokines, cells were incubated for 24 h at 37 °C and 5% CO_2_. Cell death was then measured using the WST-1 assay (Takara, Kusatsu, Shiga Prefecture, Japan) according to the manufacturer’s instructions. Absorbance was measured using an Eppendorf 2200 plate reader at a wavelength of 450 nm and a reference wavelength of 600 nm. Using WST-1 absorbance (abs), percent viability was calculated as follows: (abs cell death stimulus + ricin)/(abs neg) × 100.

### 4.4. Immunoblots

A total of 6 × 10^6^ A549 cells were seeded in a 58 cm^2^ Petri dish (Celltreat) per condition and allowed to grow for 24 h at 37 °C and 5% CO_2_. A549 cells were then treated with (1) 1 ng/mL ricin, (2) 100 ng/mL cell death ligand/cytokine (TRAIL, TNF-α, or FasL), or 3.) 1 ng/mL ricin combined with 100 ng/mL cell death ligand/cytokine for 4 h at 37 °C and 5% CO_2_. Following this, A549 cells were lysed using 1% triton-X-100 (Sigma) with 1× Halt protease inhibitor (Thermo Fisher) in 1× PBS on ice for 30 min. Cells were then sonicated on ice 3× (20 sec pulses) at an output of 10%. Cell lysates were centrifuged at 14000 rpm at 4 °C to remove nuclear material. A549 cell lysates were run on SDS-PAGE and transferred to a PVDF membrane and blocked in 1× TBS with 0.1% tween-20 and 5% milk for 30 min at room temperature. The blots were then incubated with diluted primary antibody in 1× TBS with 0.1% tween-20 and 5% milk overnight at 4 °C. All primary antibodies were obtained from Cell Signaling Technology (Danvers, MA, USA), unless otherwise indicated. Primary antibodies were used at the following dilutions: anti-human caspase-3 (1:1000), anti-human/mouse caspase-6 (1:1000), anti-human/mouse caspase-7 (1:1000), anti-human caspase-8 (1:1000), anti-human caspase-9 (1:1000), and anti-human GAPDH (1:10,000). After washing with 1X TBS with 0.1% tween-20 and 5% milk, the blots were incubated with secondary HRP-conjugate antibodies (1:5000) for 1 h at room temperature. Blots were developed by chemiluminescence and read in a Bio-Rad ChemiDoc XRS+.

### 4.5. Statistical Analyses

Statistical analyses were carried out using GraphPad Prism 7. All cell death assays were subject to statistical analysis by two-way ANOVA and Bonferroni posttest. All cell death assays are the results of 3 independent experiments. Immunoblots presented are representative of 3 independent experiments.

## Supplementary Materials

The following are available online at https://www.mdpi.com/2072-6651/11/8/450/s1, Figure S1: The combination of ricin/TRAIL induces caspase activation, Figure S2: Ricin/TNF-α and ricin/FasL do not induce caspase activation in A549 cells after 8 h, Figure S3: The apoptotic stimuli CHX/TRAIL, CHX/TNF-α, and CHX/FasL induce caspase cleavage, Figure S4: Ricin/TRAIL induces caspase cleavage that is prevented by pharmacologic inhibitors, Figure S5: A549 cell death by ricin/TNF-α and ricin/FasL does not depend on caspases-6 or -9, Figure S6: The apoptotic stimuli CHX/TNF-α and CHX/FasL induce Bax-dependent cell death, Figure S7: Cell death induced by ricin in combination with TNF-α or FasL does not depend on RIP1 kinase, Figure S8: Mcl1 protein loss does not differ between ricin/TRAIL, ricin/TNF-α, and ricin/FasL, Figure S9: Cell death induced by ricin/TNF-α and ricin/FasL does not depend on calpains or cathepsins L and K, Figure S10: The cathepsin inhibitor, CATI-1, has no effect on A549 cell death by ricin/TRAIL, Figure S11: Cell death induced by ricin/TNF-α or ricin/FasL is inhibited by N-acetylcysteine, Figure S12: Calu3 human lung epithelial cells are insensitive to cell death by ricin/TNF- or ricin/FasL, Figure S13: Calu3 human lung epithelial cells are insensitive to cell death by ricin/TNF-α or ricin/FasL,. Figure S14: Ricin/TRAIL induces caspase-dependent apoptosis while ricin/TNF-α and ricin/FasL induce cathepsin-dependent cell death in U937 human monocytes.
