# Supplementary material for: TNF Family Cytokines Induce Distinct Cell Death Modalities in the A549 Human Lung Epithelial Cell Line when Administered in Combination with Ricin Toxin
Source: Toxins (Basel). 2019 Aug 1;11(8):450. doi: 10.3390/toxins11080450 (PMC6723388; doi:10.3390/toxins11080450)
Supplement: Supplementary file 1 [file toxins-11-00450-s001.pdf]

Supplementary Materials: TNF Family Cytokines Induce Distinct Cell Death Modalities in the A549 Human Lung Epithelial Cell Line when Administered in Combination with Ricin Toxin

Alexa L. Hodges, Cody G. Kempen, William D. McCaig, Cory A. Parker, Nicholas J. Mantis and Timothy J. LaRocca

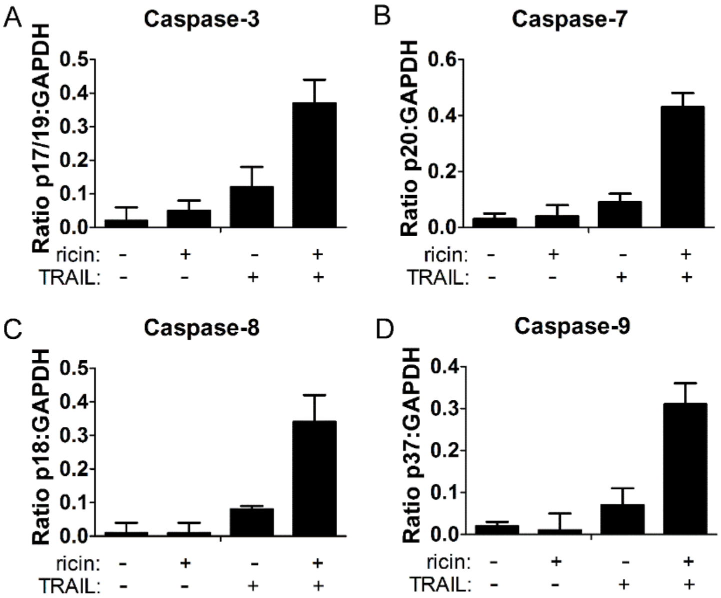

Figure S1. The combination of ricin/TRAIL induces caspase activation. A549 lung epithelial cells were treated with 1 ng/mL ricin alone or in combination with 100 ng/mL TRAIL for 4 h at 37 °C followed by cell lysis and western blot and densitometry. The combination of ricin and TRAIL results in cleavage/activation of caspases (**A**) -3, (**B**) -7, (**C**) -8, and (**D**) -9. Results shown are the average from 3 independent experiments. Error bars = standard deviation.

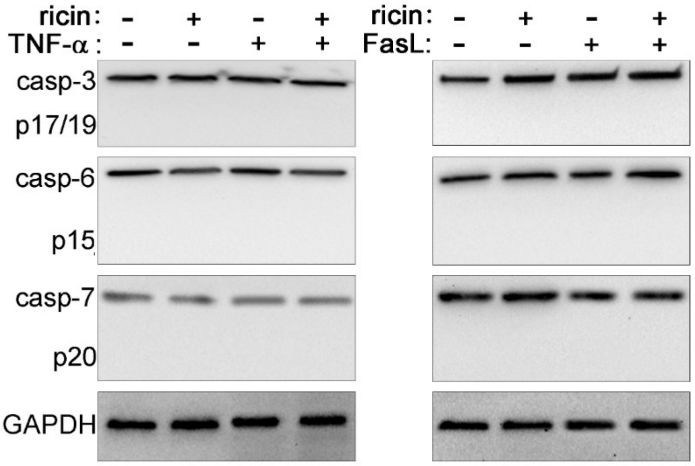

Figure S2. Ricin/TNF-α and ricin/FasL do not induce caspase activation in A549 cells after 8 h. A549 lung epithelial cells were treated with 1 ng/mL ricin alone or in combination with 100 ng/mL TNF-α or FasL for 8 h at 37 °C followed by cell lysis and western blot. The combination of ricin/TNF-α or

ricin/FasL does not result in the cleavage/activation of caspases after 8 h. Shown are representative blots from 3 independent experiments.

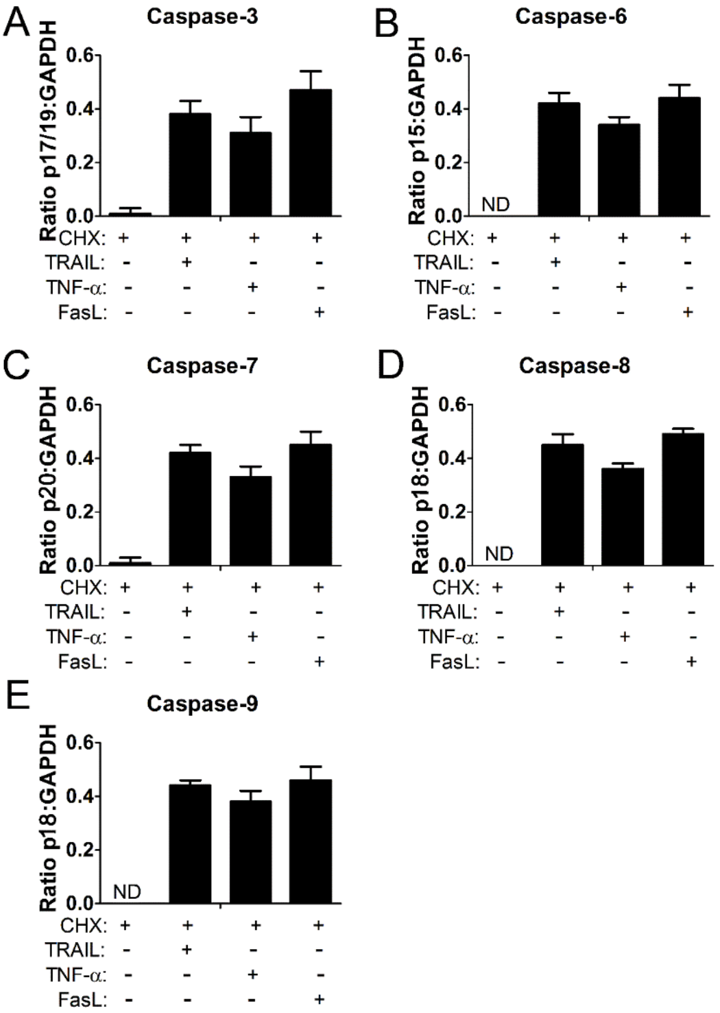

Figure S3. The apoptotic stimuli CHX/TRAIL, CHX/TNF-*α*, and CHX/FasL induce caspase cleavage. A549 cells were treated with 250 ng/mL cycloheximide (CHX) combined with TNF-*α*, FasL, or TRAIL as a positive control for TRAIL-, TNF-, and FasL-induced apoptosis. Lysates were probed on western blot and densitometry was performed. As expected, when cycloheximide is combined with TNF-*α*, FasL, or TRAIL it results in the cleavage/activation of caspases (**A**) -3, (**B**) -6, (**C**) -7, (**D**) -8, and (**E**) -9. Results shown are the average from 3 independent experiments. Error bars = standard deviation.

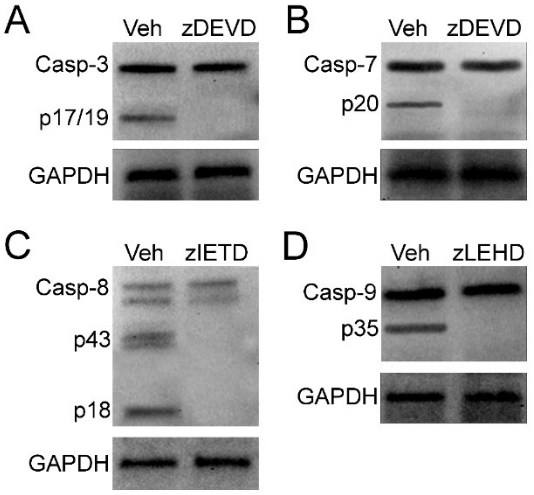

Figure S4. Ricin/TRAIL induces caspase cleavage that is prevented by pharmacologic inhibitors. A549 lung epithelial cells were treated with 1 ng/mL ricin in combination with 100 ng/mL TRAIL in the absence or presence of caspase inhibitors for 4 h at 37 °C followed by cell lysis and western blot. Ricin/TRAIL induces cleavage/activation of caspases-3, -7, -8, and -9 which is prevented by (**A**,**B**) zDEVD-fmk (30 μM), (**C**) zIETD-fmk (30 μM), and (**D**) zLEHD-fmk (10 μM), respectively. Shown are representative blots from 3 independent experiments.

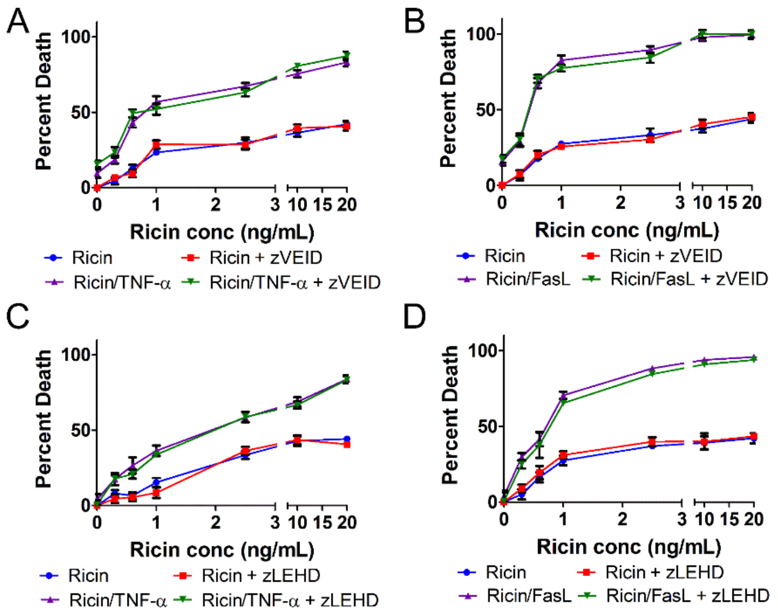

Figure S5. A549 cell death by ricin/TNF-*α* and ricin/FasL does not depend on caspases-6 or -9. A549 lung epithelial cells were treated with ricin alone or in combination with 100 ng/mL TNF-*α* in the presence or absence of caspase inhibitors for 24 h at 37 °C followed by measurement of cell death via WST-1 assay. Cell death induced by ricin/TNF-*α* or ricin/FasL was not affected by inhibition of (**A**,**B**) caspase-6 with zVEID-fmk (30 μM) or (**C**–**D**) caspase-9 with zLEHD-fmk (10 μM). Results shown are the average from 3 independent experiments. Error bars = standard deviation. Two-way ANOVA, *** $p < 0.001$.

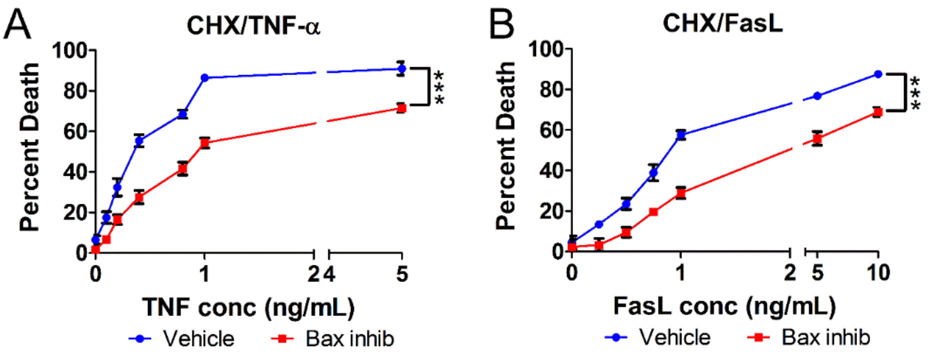

Figure S6. The apoptotic stimuli CHX/TNF-*α* and CHX/FasL induce Bax-dependent cell death. A549 cells were treated with the combination of 250 ng/mL cycloheximide (CHX) and TNF-*α* or cycloheximide and FasL as positive controls for TNF- and FasL-induced apoptosis. As expected, (**A**) cycloheximide/TNF-*α* and (**B**) cycloheximide/FasL-induced apoptosis is prevented by Bax-inhibiting peptide v5 (100 μM). Results shown are the average from 3 independent experiments. Error bars = standard deviation. Two-way ANOVA, *** $p < 0.001$.

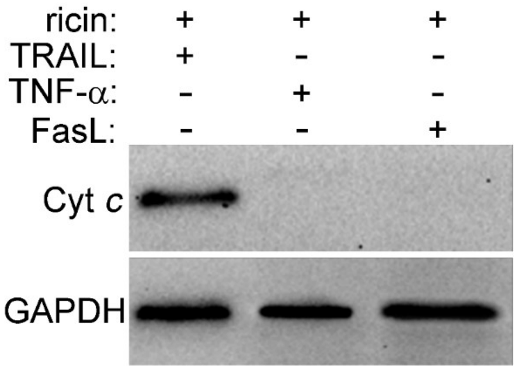

Figure S7. Cytochrome c is released into the cytoplasm of A549 cells treated with ricin/TRAIL but not those treated with ricin/TNF-*α* or ricin/FasL. A549 lung epithelial cells were treated with 1 ng/mL ricin alone or in combination with 100 ng/mL TRAIL, TNF-*α*, or FasL for 4 h at 37 °C followed by cytoplasmic extraction with digitonin and western blot. Cyt c is observed only in digitonin extracts from cells treated with ricin/TRAIL.

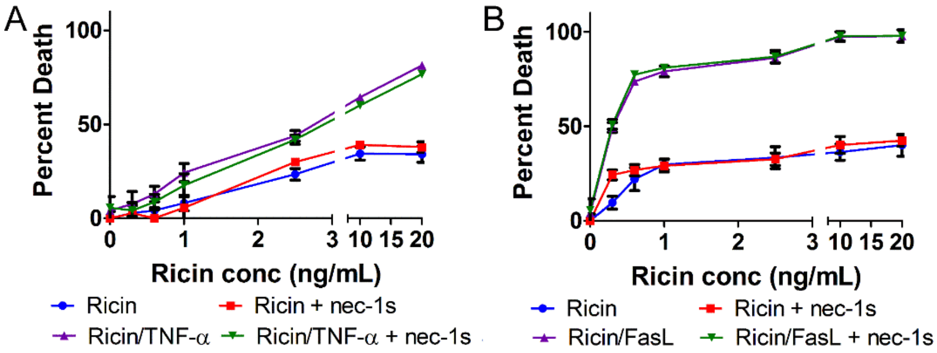

Figure S8. Cell death induced by ricin in combination with TNF-*α* or FasL does not depend on RIP1 kinase. A549 lung epithelial cells were treated with ricin alone or in combination with 100 ng/mL TNF-*α* or FasL in the presence or absence of the RIP1 inhibitior, necrostatin-1s (nec-1s, 50 μM), for 24 h at 37 °C followed by measurement of cell death via WST-1 assay. Cell death induced by the combination of ricin and (**A**) TNF-*α* or (**B**) FasL was not prevented by inhibition of RIP1. Results shown are the average from 3 independent experiments. Error bars = standard deviation. Two-way ANOVA, *** $p < 0.001$.

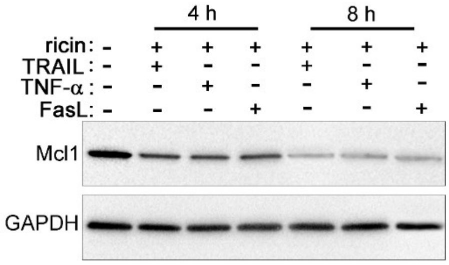

Figure S9. Mcl1 protein loss does not differ between ricin/TRAIL, ricin/TNF-*α*, and ricin/FasL. A549 lung epithelial cells were treated with 1 ng/mL ricin alone or in combination with 100 ng/mL TRAIL, TNF-*α*, or FasL for 4 or 8 h at 37 °C followed by cell lysis and western blot. Mcl1 degrades over 8 h of treatment but the pattern does not differ between ricin/TRAIL, ricin/TNF-*α*, and ricin/FasL. Shown are representative blots from 3 independent experiments.

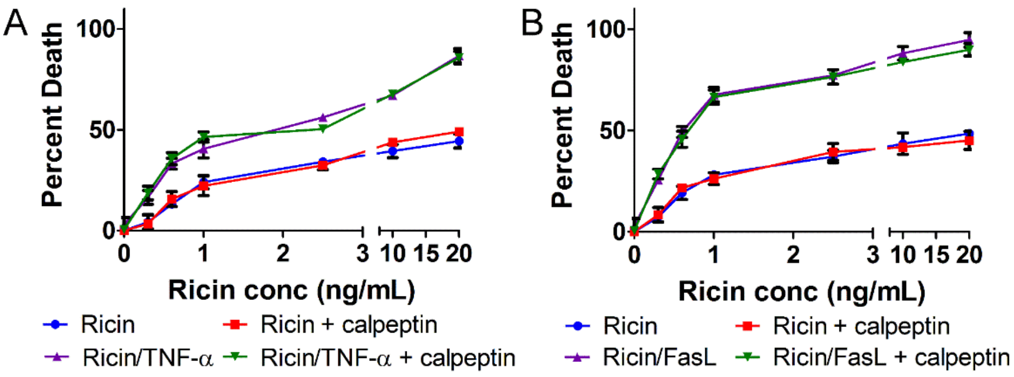

Figure S10. Cell death induced by ricin/TNF-*α* and ricin/FasL does not depend on calpains or cathepsins L and K. A549 lung epithelial cells were treated with ricin alone or in combination with 100 ng/mL TNF-*α* or FasL in the presence or absence of calpeptin (50 μM), an inhibitor of calpains as well as cathepsins L and K, for 24 h at 37 °C followed by measurement of cell death via WST-1 assay. Cell death induced by the combination of ricin and (**A**) TNF-*α* or (**B**) FasL was not prevented by calpeptin. Results shown are the average from 3 independent experiments. Error bars = standard deviation. Two-way ANOVA, *** $p < 0.001$.

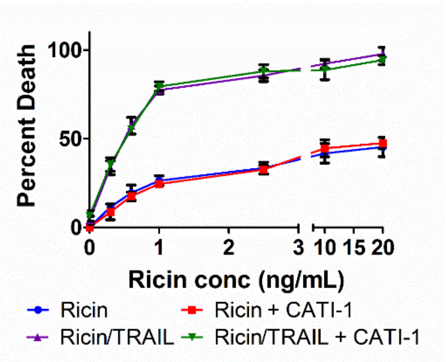

Figure S11. The cathepsin inhibitor, CATI-1, has no effect on A549 cell death by ricin/TRAIL. A549 lung epithelial cells were treated with ricin alone or in combination with 100 ng/mL TRAIL in the presence or absence of CATI-1 (20 μM) for 24 h at 37 °C followed by measurement of cell death via WST-1 assay. Cell death induced by the combination of ricin and TRAIL was not affected by inhibition of cathepsins. Results shown are the average from 3 independent experiments. Error bars = standard deviation.

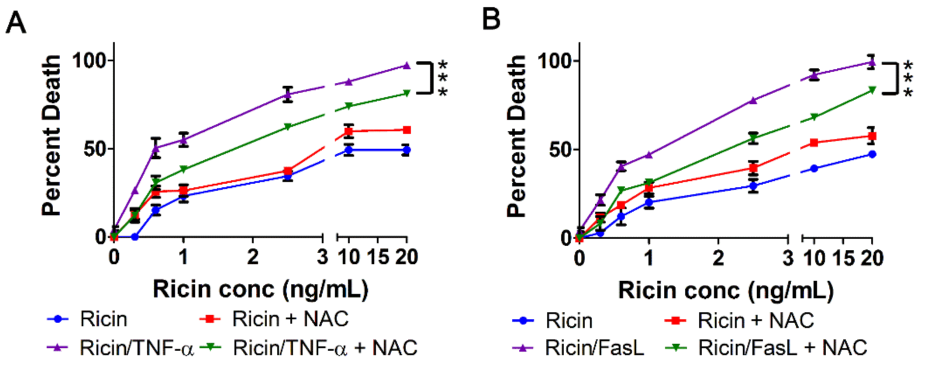

Figure S12. Cell death induced by ricin/TNF-*α* or ricin/FasL is inhibited by N-acetylcysteine. A549 lung epithelial cells were treated with ricin alone or in combination with 100 ng/mL TNF-*α* or FasL for 24 h at 37 °C followed by measurement of cell death via WST-1 assay. Cell death induced by ricin/TNF-*α* and ricin/FasL was partially prevented by inhibition of (**A**,**B**) reactive oxygen species with N-acetylcysteine (NAC, 10 mM). Results shown are the average from 3 independent experiments. Error bars = standard deviation. Two-way ANOVA, *** $p < 0.001$.

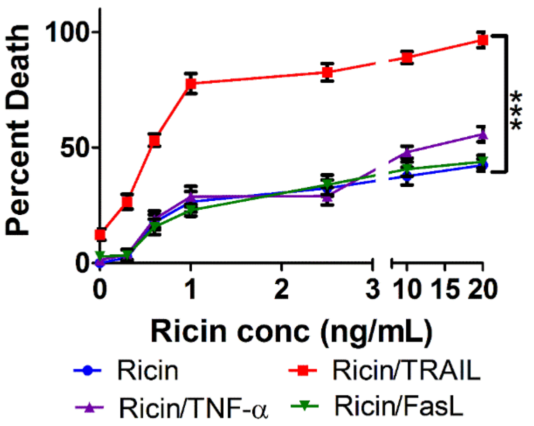

Figure S13. Calu3 human lung epithelial cells are insensitive to cell death by ricin/TNF-*α* or ricin/FasL. Calu3 lung epithelial cells were treated with ricin alone or in combination with 100 ng/mL TRAIL, TNF-*α*, or FasL for 24 h at 37 °C followed by measurement of cell death via WST-1 assay. Cell death induced by ricin/TRAIL was significantly increased relative to ricin alone. Cell death by ricin/TNF- or ricin/FasL did not increase relative to ricin alone. Results shown are the average from 3 independent experiments. Error bars = standard deviation. Two-way ANOVA, *** $p < 0.001$.

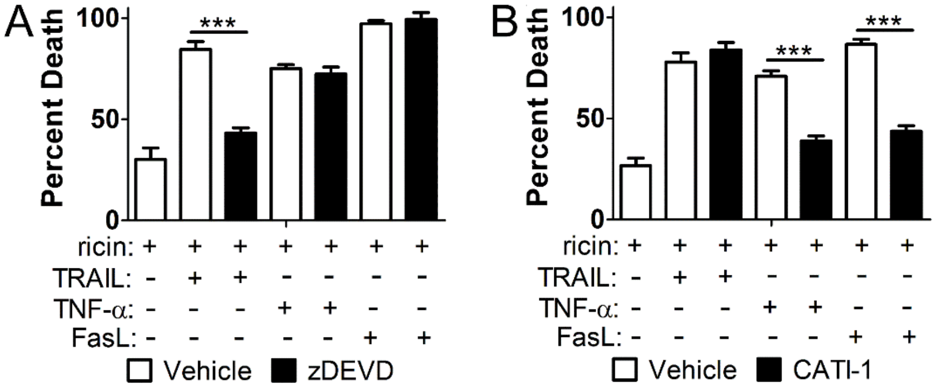

Figure S14. Ricin/TRAIL induces caspase-dependent apoptosis while ricin/TNF-*α* and ricin/FasL induce cathepsin-dependent cell death in U937 human monocytes. U937 monocytes were treated with ricin alone or in combination with 100 ng/mL TRAIL, TNF-*α*, or FasL for 24 h at 37 °C followed by measurement of cell death via WST-1 assay. (**A**) Cell death induced by ricin/TRAIL was prevented by inhibition of caspase-3 with zDEVD-fmk (30 μM). Cell death by ricin/TNF-*α* and ricin/FasL was not affected by zDEVD-fmk. (**B**) Cell death by ricin/TNF-*α* and ricin/FasL was prevented by inhibition of cathepsins with CATI-1 (20 μM). Cell death by ricin/TRAIL was not affected by CATI-1. Results shown are the average from 3 independent experiments. Error bars = standard deviation. Two-way ANOVA, *** $p < 0.001$.
